# Leveraging Comparative Genomics to Identify and Functionally Characterize Genes Associated with Sperm Phenotypes in *Python bivittatus* (Burmese Python)

**DOI:** 10.1155/2016/7505268

**Published:** 2016-04-20

**Authors:** Kristopher J. L. Irizarry, Josep Rutllant

**Affiliations:** ^1^The Applied Genomics Center, Graduate College of Biomedical Sciences, College of Veterinary Medicine, Western University of Health Sciences, 309 East Second Street, Pomona, CA 91766, USA; ^2^Molecular Reproduction Laboratory, College of Veterinary Medicine, Western University of Health Sciences, 309 East Second Street, Pomona, CA 91766, USA

## Abstract

Comparative genomics approaches provide a means of leveraging functional genomics information from a highly annotated model organism's genome (such as the mouse genome) in order to make physiological inferences about the role of genes and proteins in a less characterized organism's genome (such as the Burmese python). We employed a comparative genomics approach to produce the functional annotation of* Python bivittatus *genes encoding proteins associated with sperm phenotypes. We identify 129 gene-phenotype relationships in the python which are implicated in 10 specific sperm phenotypes. Results obtained through our systematic analysis identified subsets of python genes exhibiting associations with gene ontology annotation terms. Functional annotation data was represented in a semantic scatter plot. Together, these newly annotated* Python bivittatus *genome resources provide a high resolution framework from which the biology relating to reptile spermatogenesis, fertility, and reproduction can be further investigated. Applications of our research include (1) production of genetic diagnostics for assessing fertility in domestic and wild reptiles; (2) enhanced assisted reproduction technology for endangered and captive reptiles; and (3) novel molecular targets for biotechnology-based approaches aimed at reducing fertility and reproduction of invasive reptiles. Additional enhancements to reptile genomic resources will further enhance their value.

## 1. Introduction

Reptiles represent a diverse and biologically distinct group of vertebrates for which most species have yet to be systematically studied. Over the last few decades, reptiles in general, and snakes in particular, have grown in popularity among owners and breeders. It is worth noting that reptiles are of ecological interest as both endangered and invasive species. For example, in 2015, California Department of Fish and Wildlife listed ten distinct reptile species as either endangered or threatened, including four species of snakes:* Charina bottae* (southern rubber boa),* Thamnophis gigas* (giant garter snake),* Thamnophis sirtalis tetrataenia* (San Francisco garter snake), and* Masticophis lateralis euryxanthus* (alameda whipsnake). At the same time, California Department of Fish and Wildlife classifies other reptiles as invasive species, such as* Nerodia fasciata* (southern watersnake). The identification of genetic and genomic resources for use in reptiles can accelerate research and ultimately enhance knowledge of their unique biology. Deciphering reptile reproductive biology can provide avenues for facilitating successful breeding in endangered species and, at the same time, may offer insights into reducing the reproduction of invasive species.

Two species of the genus* Python* have recently become the focus of genomics level investigations. Castoe et al. sequenced the genome of the Burmese python as a reptilian model organism with the expectation that the sequence data would provide insight into unique aspects of reptilian physiology and evolution [1]. Similarly, whole transcriptome studies in ball python have led to the production of genomics resources which can be used to further study this species [2]. Recent investigations into python physiology have identified rapid gene expression changes associated with basic physiological processes such as eating [3]. For example, the Burmese python exhibits unique organ and metabolic adaptations which have been characterized recently at the molecular and genetic levels [4].

*Python regius *(ball python or royal python) is a relatively small member of the Python family which has become an extremely popular pet over the last 15 to 20 years due to the tremendous expansion of color variations that have been produced.* P. regius* is a relatively small species in length (rarely over 2 meters) and adapts easily to being raised in captivity. The name “ball python” is derived from the fact that this species curls up in a ball whenever it is approached or handled, making it particularly easy to manage in captivity.

*Python molurus bivittatus* (Burmese python) has been a popular pet in the United States since the 1990s due to their attractive color patterns, docile nature, and large size [5]. Size-wise, the Burmese python is one of the largest snakes in the world and can reach over 23 feet in length and weights of over 200 pounds. Some of these giant snakes have been illegally released by their owners into the wild due to the difficulty to handle them and the lack of alternate housing or sheltering. Nowadays, although native to Southeast Asia, these snakes are exotic (nonnative) species in areas like South Florida (e.g., Everglades National Park) and they are also considered invasive species [6] since they are not constrained by natural factors. Consequently, due to their potential to harm invaded environments (wildlife and ecosystem), efforts are underway to reduce their numbers in these sensitive environments. Because of their large size, Burmese pythons have few predators, and subsequently their predation upon native species is decreasing the native populations to the level of being threatened or endangered [7, 8]. The impact of the invasive Burmese pythons on the normal wild life is serious concern. Dorcas et al. describe the severe reduction in mammals in the Florida Everglades due to the pythons growing population size [9]. Novel genetic strategies are being employed to monitor the Burmese python. Recently, PCR-based detection methods have been employed to detect Burmese python DNA in environmental water samples, such as marshes, streams, swamps, and lakes [10].

In contrast to invasive reptiles, endangered reptiles are exhibiting decreased numbers in their native ecosystems. Populations of endangered reptiles may suffer from inbreeding due to reduced populations or even reproduction in a captive setting such as a zoo [11]. Hussain et al. developed methods to quantify damage to semen at specific steps in the preservation process [12]. The work was carried out in mammals; however, the approach is viable for reptiles as well. Ruiz-Lopez et al. describe the relationship between homozygosity, heterozygosity, and inbreeding depression [13]. These undesirable genetic issues arise when populations are endangered and can contribute to decreased reproductive fitness, including poor sperm function, reduced motility, and decreased sperm numbers in the endangered population. Birds have been used as models for developing reproductive technology such as artificial insemination and extenders capable of improving the longevity and value of cryopreserved semen from endangered species. For example, a 2009 study evaluated post-thaw semen quality in wild-caught Griffon vultures and determined that cryopreservation of semen is a useful tool in the conservation of endangered species genetic resources [14].

Assisted reproductive technology helped immensely to improve genetic pools in farm and domestic animals for several decades; however, although recognized as an important strategy to enhance diversity and increase captive populations of endangered animals, these techniques are rarely applied to reptiles [15] and much less to the specific field of snakes [16]. Reproduction in snakes differs from mammalian reproduction in many distinct ways, but the most significant difference is that spermatozoa can be stored in the female genital tract for months, if not years, before fertilization [17]. Studies related to the development of assisted reproductive techniques in snakes have been ignored and only few reports on semen collection [18, 19], sperm preservation [20], and artificial insemination [15] have been the focus of large research efforts.

Comparative genomics has been successfully employed in previous studies to identify physiologically important genes in one organism based on the annotation provided by a model organism genome. The comparative genomics approach was developed and heavily leveraged in the 1990s to facilitate functional annotation of large-scale human EST data produced during the effort to sequence the human genome [21]. Functional information about gene-phenotype relationships in model organisms were shown to be extremely valuable in deciphering the consequence of identified mutations in human genes [22]. Comparative genomics approaches became more widespread following the sequencing of multiple genomes and the emerging need to characterize unknown genes [23]. For example, genes sequenced from hamster testis were used to identify and characterize genes previously uncharacterized in human, mouse, rat, and pufferfish genomes [24]. Beyond simply facilitating the identification of novel genes, comparative genomics approaches have been effectively used to characterize individual proteins involved in sperm phenotypes, such as the sperm mitochondrial cysteine-rich protein, SMCP [25]. Such approaches have also been effective in identifying economically important reproductive traits in agriculturally important species, such as the pig [26]. Similarly, the characterization of 1227 genes in the domestic cat was achieved using a comparative genomics approach in which cat genes having phenotypically characterized orthologs in the mouse were annotated with developmental, clinical, and nutritional phenotypes [27].

A number of bioinformatics resources have been developed to facilitate the use of comparative and functional genomics. Ontologies, which are controlled vocabularies organized around specific parent-sibling relationships and maintained in a graph structure, provide standardized nomenclature and relationships among biologically relevant terminology for applications in bioinformatics and functional genomics [28]. One of the most widely used ontologies in genomics is the gene ontology [29]. Gene set enrichment, such as the identification of shared biological processes among a set of differentially regulated genes in a gene expression experiment, represents one of the most widely used applications of gene ontology [30]. Gene set enrichment has been previously applied to the identification and analysis of genes associated with phenotypes [14, 31]. Other ontologies have been developed that are useful for functional annotation of genes, including the mammalian phenotype ontology [32], the human phenotype ontology [33], and the mouse-human anatomy ontology [34].

In addition to ontological resources, pathway mapping resources provide additional means of functionally annotating genes based on biologically relevant information. One of the most widely utilized pathway resources for gene annotation is the KEGG pathway database [35] which provides knowledge-based representations of biochemical pathways and protein interaction networks. Tools have been developed that facilitate the identification of KEGG pathway members from a set of genes [36, 37]. One of the most widely used tools, based on more than 21,000 citations, is the Database for Annotation, Visualization, and Integrated Discovery [38] that provides an interface for functional gene annotation including gene ontology and KEGG pathway analysis.

**Figure 1:**
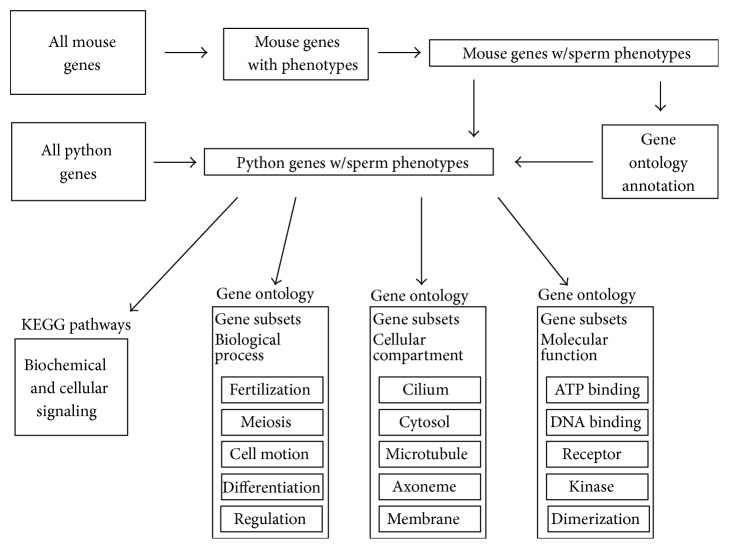
Overview of the comparative genomics approach to identify and characterize python genes associated with sperm phenotypes. The set of mouse protein coding genes was used to select the subset of mouse genes for which phenotype annotation information was available. Starting with all mouse genes having phenotype annotation, we identified the subset corresponding to protein coding genes associated with only sperm phenotypes. This set of mouse protein sequences was subsequently used to identify the corresponding protein coding sequences in* Python bivittatus *(i.e., orthologous genes). For each protein coding sequence shared between mouse and python, a pairwise protein sequence alignment was generated and measures of sequence identity and significance were calculated. Gene ontology (GO) annotation provides gene level information about biological processes, cellular locations, and molecular functions of gene products. The existing GO annotation for each mouse gene was “added” to each python orthologous gene. Then set of python genes was analyzed for statistically significant enrichment of genes associated with particular GO annotation terms across the three GO categories (biological process, cellular component, and molecular function). This resulting set of annotated python genes provides additional biological, physiological, cellular, and molecular information about the roles of these genes in sperm production and function. Moreover, the annotation also offers an independent set of annotation information to help validate the python genes as truly being associated with sperm biology. Additionally, cellular pathways which are associated with the sperm associated gene set were identified along with human disorders caused by human orthologs of these genes.

The relatively recent production of reptile genomic resources has opened the door to leveraging genome-scale information to address the challenges associated with endangered and invasive reptiles. In an attempt to expand the set of genetic resources available for investigating reptile biology, we have chosen to utilize a comparative genomics strategy to identify python genes likely to play a significant role in sperm development and function (Figure 1). We describe the selection of a set of mouse genes that have been previously demonstrated to modulate sperm phenotypes and use that mouse set to identify and functionally characterize a corresponding set of python genes. Although reptiles and mammals share many aspects of biology with each other, they each also have evolved unique adaptations that are found only in their respective lineages. By leveraging mammalian reproductive genetics to initiate the construction of reptile reproductive genomics tools, the long and productive history of mouse biology and genetics can be brought to bear on the emerging field of reptile reproductive genetics.

## 2. Materials and Methods

### 2.1. Comparative Genomics Approach to Identify Python Genes Associated with Sperm Phenotypes

The comparative genomics approach (Figure 1) leveraged the set of mouse genes for which phenotype information was available. Mouse genes annotated with sperm specific phenotypes were identified and python orthologs were identified as described below. Gene ontology enrichment was performed to identify gene ontology biological process, cell component, and molecular function terms associated with each gene set identified by a sperm associated phenotype. The resulting set of python genes mapped to sperm phenotypes is available as a supplemental data file, as is a second supplemental file containing the mapping of the mouse genes to the corresponding python orthologs, all of which are mapped to sperm phenotypes.

### 2.2. Identification of* P. regius* and* P. bivittatus* Sequences from NCBI

Publicly available DNA, mRNA, and protein* Python bivittatus* sequences were downloaded from the NCBI nucleotide and protein sequence databases (http://www.ncbi.nlm.nih.gov/) by searching NCBI for “Python bivittatus.” A total of 25,944 protein sequences were identified.* P. regius *sequences were also downloaded from NCBI using a similar query for which “Python regius” was used as search term. Although considerable sequence data exists for* P. bivittatus* (20,392 genes, 25,944 protein sequences, and 105,311 nucleotide sequences), relatively few sequence resources are available for* P. regius* (21 genes, 141 protein sequences, and 123 nucleotide sequences). Interestingly, some of the identified* P. regius* sequences in the database correspond to viral sequences, such as the ball python nidovirus (8 genes, 3 nucleotide sequences, and 17 protein sequences). Even so, we identified a set of* P. regius *sequences for which orthologous* P. bivittatus *sequences were available. Because we are specifically interested in python molecular reproductive genetics, we selected (from among the set of 21 genes) those for which published papers had previously implicated the gene as (1) involved in sperm related biology or (2) being associated with variation in sperm parameters, or (3) being associated with variation in fertility in another species (Table 1).

### 2.3. Selection of Mouse Genes Associated with Sperm Phenotypes

Mouse gene and protein identifiers along with protein sequences were downloaded from the Ensembl database (http://www.ensembl.org/). Data was loaded into a table in a relational database (MySQL) containing phenotype annotation from the Mammalian Phenotype Browser (http://www.informatics.jax.org/searches/MP_form.shtml). Database queries in SQL provided a mechanism for selecting all mouse gene identifiers linked to specific phenotypes. The output from the database contained individual gene-phenotype relationships. Since a single gene could be associated with more than one phenotype, the results contained unique gene-phenotype relationships even though the same genes were listed under multiple phenotypes. The list of 129 sperm related phenotypes mapped to each mouse gene (listed as mouse ensemble gene identifier) is contained in Supplemental File 1 (see Supplemental File 1 in Supplementary Material available online at http://dx.doi.org/10.1155/2016/7505268).

### 2.4. Mapping Mouse Genes Associated with Sperm Phenotypes to* P. bivittatus* Protein Identifiers

Each mouse gene ID was used to query the database to retrieve the gene description field which contains a variety of header information including the complete title of the gene. The mouse gene title information was used to manually search through NCBI's protein database (http://www.ncbi.nlm.nih.gov/protein/?term=Python%20bivittatus) for orthologous python protein sequences. Because the goal of the project was to insure the collection of genes having sperm phenotypes, any genes which were not the obvious ortholog of the mouse gene were excluded from the final dataset. For example, in some cases, a specific family member of a multigene family was sought, but the exact family member could not be found even though a number of paralogous* P. bivittatus* genes from the same family were located.

Every successfully identified* P. bivittatus* gene was downloaded and stored in FASTA format for later use. Once the complete set of mouse gene identifiers was mapped to all available python orthologs, a file containing the complete set of snake protein sequences was used to develop a blast database using the* formatdb* tool with the –p option set to true to indicate amino-acid sequences versus nucleotide sequences.

Finally, the blastall program was used to perform blastp analysis of the protein sequences associated with sperm phenotypes. For each orthologous mouse-python gene pair, blastp calculated the bit score, generated an alignment, and calculated the percent identity. A tab-delimited text file containing each sperm associated phenotype, the Ensembl mouse gene identifier, official gene symbol, the Burmese python NCBI gene identifier, the Burmese python NCBI refId, and the description of each gene is contained in Supplemental File 1.

### 2.5. Pairwise Alignments, Multiple Sequence Alignments, and Phylogenetic Trees

**Figure 2:**
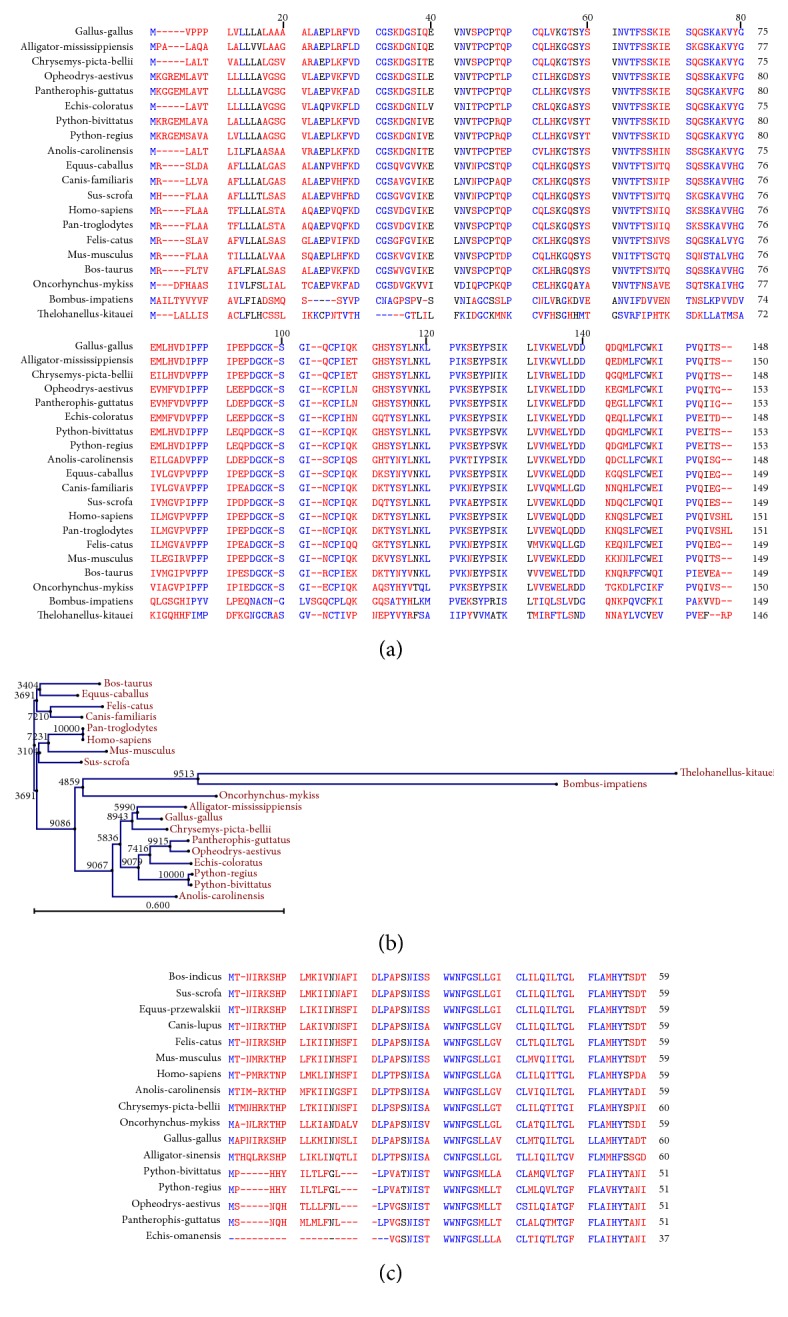
(a) Multiple sequence alignment of epididymal protein E1. Twenty species representing fish, parasite, insect, snake, turtle, lizard, alligator, chicken, mouse, cow, horse, dog, cat, chimpanzee, and human conserved regions of the alignment are indicated in blue while nonconserved regions are shown in red. Sequences from the snake species* Opheodrys aestivus*,* Pantherophis guttatus*,* Echis coloratus*,* Python regius*, and* Python bivittatus *all contain a 5-amino-acid insertion (KRGEM in python species) within the first ten amino acids of the alignment in comparison to other reptiles and mammalian species. Similarly, the primate lineage, represented by* Homo sapiens *and* Pan troglodytes*, exhibits a 2-amino-acid insertion (HL) at the very end of the alignment.* Thelohanellus kitauei *(an aquatic invertebrate parasite) and* Bombus impatiens* (bee) provide evidence for an ancient role of this gene in the common ancestor of insects, parasites, and vertebrates. (b) Phylogenetic tree of epididymal protein E1. The multiple sequence alignment shown in Figure 3(a) was used to generate a phylogenetic tree using a bootstrapping approach. The number near each root or interior node of the tree indicates how many times the same subtree, as shown in the image, was obtained when the input sequences were sampled during the bootstrapping. In this tree,* Python regius *and* Python bivittatus* are grouped together in all 10,000 trees produced during the phylogenetic tree construction process. Similarly, the* Homo sapiens *and* Pan troglodytes *subtree also exhibits a count of 10000. The scale bar below the tree provides an estimate for sequence evolution rate between the taxa. (c) Multiple sequence alignment of mitochondrial cytochrome b protein. The cytochrome b protein was aligned across multiple species to visualize the sequence relationship between* Python regius *and* Python bivittatus *in the context of other species. Conserved regions of the alignment are indicated in blue while less conserved regions are shown in red. The species representing snakes exhibit an absence of the 5 amino acids within the first ten amino acids of the alignment. This sequence feature is not observed in other reptiles,* Gallus gallus *(chicken) and* Oncorhynchus mykiss *(trout), or in mammals. Thus, there is the possibility that certain aspects of biology and/or physiology may be uniquely shared among snakes.

Epididymal protein E1 sequence was obtained for the following species:* Gallus gallus* (chicken),* Alligator mississippiensis* (alligator),* Chrysemys picta bellii* (painted turtle),* Pantherophis guttatus* (corn snake),* Echis coloratus* (palestine saw-scaled viper),* Python bivittatus* (burmese python),* Python regius* (ball python),* Anolis carolinensis* (Carolina anole),* Equus caballus* (domestic horse),* Canis familiaris*,* Sus scrofa*,* Homo sapiens*,* Pan troglodytes*,* Felis catus*,* Mus musculus* (mouse),* Bos taurus* (cow),* Oncorhynchus mykiss* (trout),* Bombus impatiens* (eastern bumble bee),* Thelohanellus kitauei* (fish parasite). The mitochondrial cytochrome b protein sequence was obtained for multiple sequence alignment across 17 species (Figure 2(c)). The equine sequence was taken from* Equus przewalskii*, the feral horse, instead of the domestic horse. Alignments were generated using CLC Sequence Viewer 6 (http://www.clcbio.com/products/clc-sequence-viewer/). Phylogenetic trees were produced using the tree construction algorithm in CLC Viewer 6.

### 2.6. Functional Gene Ontology Enrichment Analysis of Genes Associated with Sperm Phenotypes

Gene set analysis was performed on the set of 98 genes associated with phenotypes using the online bioinformatics package DAVID (https://david.ncifcrf.gov/). The DAVID bioinformatics resource provides genome level functional annotation of genes and data sets through a web-based interface [52]. It is an ideal resource that facilitates the identification of biologically relevant signals in large-scale genomics data sets [53].

Mapping of mouse gene identifiers to python protein identifiers provided a means to accomplish analysis of the python genes using the DAVID mouse gene identifiers since the DAVID database relies upon established gene ontology associations with gene identifiers. At the time of writing, the DAVID database does not handle analyses using python gene or protein identifiers. Running the functional genomic analysis using the mouse gene identifiers as surrogates for the orthologous python genes enabled the gene ontology analysis to be accomplished with the DAVID software.

Gene ontology enrichment terms were identified using a *p* value <0.05 and/or Benjamini probability <0.05. Because the purpose of the DAVID software suite is to identify biological annotations that are enriched in gene sets from gene expression studies, it is of value to use stringent statistical measures of enrichment. However, unlike a gene expression experiment, the genes identified in this study each are selected due to their individual phenotype relating to sperm development, function, and morphology. Therefore, even in cases where the *p* value is >0.05, the functional annotation associated with a specific gene is still the true biology underlying that gene's involvement in reproductive biology.

### 2.7. Characterization of Phenotype-Specific Functional Annotation in Python Sperm Genes

Additional rounds of gene set functional analysis were employed to phenotype-specific biological annotations. Specifically, gene ontology enrichment terms were identified using a *p* value <0.05 and/or Benjamini probability < 0.05 for each of the ten sperm related phenotypes. The analysis was carried out using the DAVID bioinformatics software tool. Gene sets from each of the ten sperm phenotypes were analyzed and the results were compared to the functional annotation identified in the 98-gene set. As was considered in the analysis of the 98-gene set, annotation terms relating to reproduction and fertility were included in the functional annotation of genes even in cases where the *p* value was >0.05.

### 2.8. Visualization of Phenotype-Specific Functional Annotation in Python Sperm Genes

Visualization of gene ontology annotation was accomplished using Revigo (Supek 2011) which creates two-dimensional scatterplots and tree maps that organize the annotations via their semantic relationships with one another. The gene ontology* biological process*,* cellular compartment*, and* molecular function* terms identified via the characterization of phenotype-specific annotation were used as input for Revigo. The *p* values were converted to −log(*p*  value) and included as scores for each GO annotation. Parameters were set to reflect that −log(*p*  value) entered as the score with the GO annotation was considered higher scoring if the associated −log(*p*  value) was higher (this is in contrast to *p* values in which lower values are better scores). Additionally, the analysis was performed using the GO database from* Homo sapiens* because it contained the greatest number of genes from the set of sperm associated phenotypes (97 out of 98) compared to* Mus musculus* and* Gallus gallus*. Since enrichment metrics utilize the hypergeometric distribution for calculating statistical significance,* H. sapiens* was considered more appropriate based on inclusion of sperm associated genes represented in the Revigo resource. The SimRel measure of semantic similarity was used for the analysis.

### 2.9. KEGG Pathway Enrichment Analysis of Genes Associated with Sperm Phenotypes

KEGG database provides a repository for genes associated with cellular and signaling pathways [54] which can be used to decipher gene functions. Pathway enrichment analysis was performed on the set of 98 genes associated with sperm phenotypes using the online bioinformatics package DAVID (https://david.ncifcrf.gov/) [55, 56].

### 2.10. Public Release of Data and Functional Annotation Associated with This Study

The authors of this project believe that the benefit of genomics and genetic resources is best accomplished when such resources are freely made available to the research community. Subsequently, the set of mouse and python gene identifiers, along with their orthologous mappings and functional associations with specific phenotypes, have been made freely available to the research community through the supplemental data associated with this publication (Supplemental File 1 and Supplemental File 2). Specifically these supplemental resources include tab-delimited files with gene ontology (biological process, cellular compartment, and molecular function) enrichment results for the sperm associated genes (Supplemental File 3, Supplemental File 4, and Supplemental File 5, resp.) as well as a FASTA file containing the protein sequences for* Python bivittatus *along with the corresponding sperm associated phenotype for each sequence (Supplemental File 6).

## 3. Results

### 3.1. Analysis of Protein Sequence Identity between* Python regius* and* Python bivittatus*

To explore the possibility that* P. regius* and* P. bivittatus *exhibit sufficient genetic similarity to justify using one species as a genetic model for the other species, we assessed the level of sequence identity among a set of protein coding sequences, mitochondrial DNA sequences, and protein sequences. We aligned the mRNA for epididymal protein E1 and assessed the extent of identity between the two species. The mRNAs were aligned using pairwise nucleotide BLASTN resulting in an alignment length of 1213 nucleotides with a percent identity of 98% corresponding to 1189 identities with 6 gaps. This alignment produced a bit score of 2102 and an *e*-value of 0. Pairwise BLASTP was used to assess the identity for the pairwise protein alignment for epididymal protein E1 between the two python species. The length of protein sequence was 153 amino acids long in both species. The aligned sequences covered the full length of each protein with an identity of 98% corresponding to 151 identical amino acids aligned with 0 gaps. A set of 12 proteins implicated in sperm related functions were selected for pairwise alignment between* P. regius* and* P*.* bivittatus* orthologs in order to assess the extent of protein sequence identity between the two species (Table 1). Together these orthologous alignments provide insight into the genetic relationship between these two python species. Four out of the twelve genes identified exhibited identity of 97% or greater (epididymal secretory protein E1, Kallikrein, Cystatin F, and cathepsin D), while just two ortholog pairs have percent identity below 90% (alpha enolase having 88% and mitochondrial NADH dehydrogenase subunit 4 with 89%).

### 3.2. Multispecies Sequence Alignments and Construction of Phylogenetic Trees across Taxa

To gain a better appreciation for the relationship that exists among taxa, with regard to the proteins implicated in sperm function, multiple sequence analysis was performed for two protein coding sequences: epididymal protein E1 (Figures 2(a) and 2(b)) and mitochondrial cytochrome b (Figure 2(c)). Sequences from the snake species (*Opheodrys aestivus*,* Pantherophis guttatus*,* Echis coloratus*,* Python regius*, and* Python bivittatus*) all contain a 5-amino-acid insertion (KRGEM in python species) within the first ten amino acids of the epididymal protein E1 alignment (Figure 2(a)) in comparison to other reptiles and mammalian species. Similarly, the primate lineage, represented by* Homo sapiens* and* Pan troglodytes,* exhibits a 2-amino-acid insertion (HL) at the C-terminal end of the alignment (Figure 2(a)).* Thelohanellus kitauei *(an aquatic invertebrate parasite) and* Bombus impatiens* (bee) provide evidence for an ancient role of this protein coding gene in the common ancestor of insects, parasites, and vertebrates. The multiple sequence alignment shown in Figure 2(a) was used to generate a phylogenetic tree using a bootstrapping approach (Figure 2(b)). The number near each root or interior node of the tree indicates how many times the same subtree, as shown in the image, was obtained when the input sequences were sampled during the bootstrapping. Larger numbers indicate a greater percent of bootstrapped trees contained in the same tree organization as depicted in the figure.* Python regius* and* Python bivittatus* are grouped together in all 10,000 trees produced during the phylogenetic bootstrapping tree construction process. The only other species, for which 10,000 iterations of tree construction resulted in the two species being paired together 100% of the time, are* Homo sapiens* and* Pan troglodytes*. The common evolutionary relationship between the snake species exhibiting the 5-amino-acid insertion at the beginning of the alignment is also characterized by the magnitude of the number at the subnode of the tree which contains these species.

The mitochondrial cytochrome b protein sequence was obtained for multiple sequence alignment across 17 species (Figure 2(c)). The equine sequence was taken from* Equus przewalskii*, the feral horse, instead of the domestic horse. Upon inspecting the alignment, it was apparent that the snake sequences diverged from the nonsnake species. However, in the case of mitochondrial cytochrome b, the snakes (*P. bivittatus*,* P. regius*,* P. guttatus*, and* E. omanensis*) exhibit two short deletions within the first 30 amino acids of the alignment, in contrast to the insertion identified in epididymal protein E1. Although sequence divergence within these regions of the alignment is evident among the other species, neither the nonsnake reptiles nor the mammals exhibit the gapped alignment pattern observed in the snakes.

### 3.3. Identification of* P. bivittatus* Protein Sequences Associated with Sperm Phenotypes

Through the comparative genomics approach employed, 129 gene-phenotype relationships were identified in* P. bivittatus *genes (Supplemental File 1). Initially we sought to identify 152 gene-phenotype relationships based on the phenotype annotation in the mouse. However, while attempting to identify orthologous genes in the python, 13 orthologs could not be adequately identified due to ambiguity in resolving whether some python genes were truly the orthologs, or whether what was identified in the database was a paralogous sequence.

In some cases, the identified python gene contained the annotation term “partial” in the fasta header line in NCBI databases. These sequences were still included in the final gene set (even though the sequence may not be complete). Python genes lacking the term “partial” were considered to be full length; however during our analysis it became apparent that some genes lacking the annotation “partial” did not represent full length sequences.

**Figure 3:**
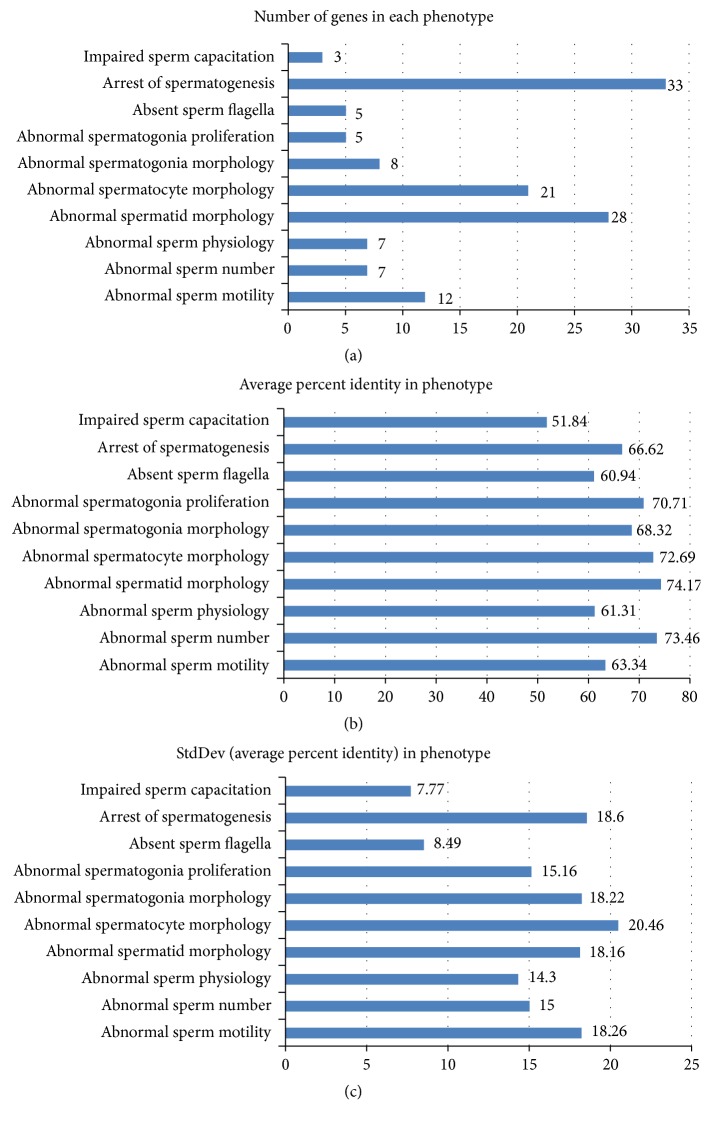
(a) Number of genes in each phenotype. The number of genes in each phenotype is shown in histogram. Within each phenotype, no gene is duplicated. However, a gene may appear in more than one phenotype. The sum of the counts is 129 corresponding to 129 gene-phenotype relationships. The number of distinct genes is 98. (b) Average percent identity for the set of genes within each phenotype. The average percent identity was calculated for each phenotype. The percent identity for each* P. bivittatus *protein sequence was determined by using BLASTP to align each* P. bivittatus *protein sequence against its corresponding mouse protein ortholog. Only the single top scoring blast hit was used to determine identity. Average identity was calculated for each phenotype by summing the individual identities within each phenotype and dividing by the total number of genes within each phenotype. (c) Standard deviation of the “average percent identity” in each phenotype. The population standard deviation of percent identity was calculated for each phenotype. The standard deviations range from a low of 7.7 to a high of 20.46. The two phenotypes with the lowest standard deviations are “impaired sperm capacitation” and “absent sperm flagella.”

The final set of* P. bivittatus* gene sequences associated with sperm phenotypes included 98 distinct genes (Supplemental File 2) mapping to ten classes of phenotype (Supplemental File 6). In order to carefully maintain the relationship between phenotype and gene, our approach treated each gene-phenotype relationship as a unique data point. Subsequently the 129 gene-phenotype relationships collapsed down to 98 distinct genes once duplicate genes were excluded. The number of* P. bivittatus* genes associated with each phenotype is shown in Figure 3(a). The average percent identity for each phenotype is shown in Figure 3(b) and the standard deviation for the average percent identity within each phenotype is shown in Figure 3(c).

**Figure 4:**
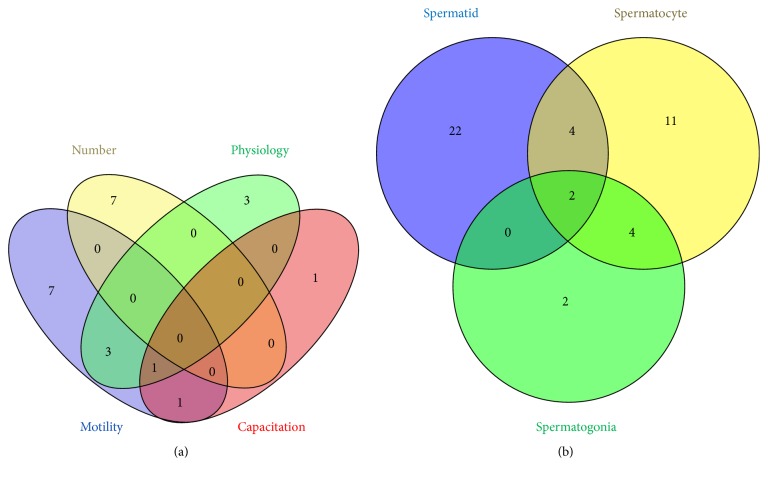
(a) Venn diagram illustrating relationship of genes among phenotypes associated with mature sperm. Genes in each of the four mature sperm phenotypes gene sets (sperm number [7 genes], sperm motility [12 genes], sperm physiology [7 genes], and capacitation [3 genes]) were analyzed for overlap across the phenotypes. A total of 23 unique genes were distributed among the phenotypes with 7 genes being exclusive to sperm motility, an additional 7 genes were unique to the sperm number phenotype, 3 genes were specific to sperm physiology, and just a single gene was only associated with capacitation. Three genes were common between sperm motility and sperm physiology while just a single gene was found to be associated with both motility and capacitation. Interestingly, one gene was associated with the motility, physiology, and capacitation phenotypes. The majority of genes were unique to specific phenotypes. (b) Venn diagram illustrating relationship of genes among phenotypes associated with morphological phenotypes in sperm precursors. Genes in each of the three abnormal morphological phenotypes associated with spermatogonia [8 genes], spermatocytes [21 genes], and spermatids [28 genes] were analyzed for overlap across the distinct phenotypes. A total of 45 genes were distributed among the phenotypes with 22 unique to spermatids, 11 unique to spermatocytes, and just 2 genes unique to spermatogonia. Four genes were common among spermatids and spermatocytes while another four genes were common spermatocytes and spermatogonia. Only two genes were associated with all three phenotypes.

Genes in each of the four mature sperm phenotypes gene sets (sperm number [7 genes], sperm motility [12 genes], sperm physiology [7 genes], and capacitation [3 genes]) were analyzed for overlap across the phenotypes (Figure 4(a)). A total of 23 unique genes were distributed among the phenotypes with 7 genes being exclusive to sperm motility, an additional 7 genes were unique to the sperm number phenotype, 3 genes were specific to sperm physiology, and a single gene was associated with capacitation. Three genes were common between sperm motility and sperm physiology while just a single gene was found to be associated with both motility and capacitation. Interestingly, one gene was associated with the motility, physiology, and capacitation phenotypes. The majority of genes were unique to specific phenotypes.

Genes in each of the three abnormal morphological phenotypes associated with spermatogonia [8 genes], spermatocytes [21 genes], and spermatids [28 genes] were analyzed for overlap across the distinct phenotypes (Figure 4(b)). A total of 45 genes were distributed among the phenotypes with 22 unique to spermatids, 11 unique to spermatocytes, and just 2 genes unique to spermatogonia. Four genes were common among spermatids and spermatocytes while another four genes were common spermatocytes and spermatogonia. Only two genes were associated with all three phenotypes.

### 3.4. Functional Analysis of Sperm Phenotype-Specific Gene Sets Using Gene Ontology Annotation

**Figure 5:**
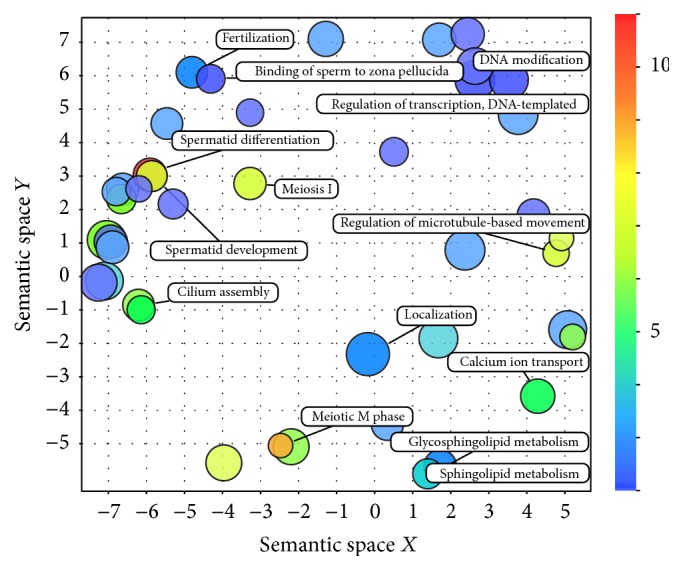
Semantic space scatter plot of gene ontology biological process annotation (node labels minimized). Gene ontology (GO) biological process annotations associated with subsets of genes within each sperm associated phenotype were visualized using a two-dimensional semantic space scatterplot. The spatial organization of annotations is based on semantic similarity. The number of node labels is minimized to allow visualization of the node colors on the scatterplot. The score = −log⁡(*p* value) for each GO annotation term node. Blue nodes indicate less significant *p* values and red nodes indicate more significant *p* values.

Gene ontology (GO) enrichment was performed to assess the biological role of the sperm associated python genes (Table 2). Among biological process annotation, highly significant terms were identified relating to reproduction including “gamete generation”, “spermatogenesis”, “germ cell development”, “spermatid differentiation”, and “meiosis”. Many of these terms were associated with *p* values as low as 7.20*E* − 40 and 7.70*E* − 37. Among the enriched GO terms representing cellular component information were cilium, cell projection, acrosomal vesicle, and microtubule cytoskeleton. Within the molecular function GO terms enriched themes of transcriptional factor regulation and DNA binding were identified as well as ATP binding and kinase activity. The complete set of GO annotation data, including a list of genes enriched for each identified GO annotation term, is available in Supplemental File 3 (biological process), Supplemental File 4 (cellular compartment), and Supplemental File 5 (molecular function). A two-dimensional semantic scatter plot was generated from the gene ontology biological process annotation (Figure 5) in order to facilitate visualization of the GO enrichment data. Semantic relationships within the gene ontology annotation terms provide evidence of common themes relating to spermatogenesis and sperm motility and function in the context of reproduction.

### 3.5. KEGG Pathways Enriched for* P. bivittatus* Genes Associated with Sperm Phenotypes

KEGG pathways enriched for genes within the set of 98* Python bivittatus *genes associated with sperm phenotypes were identified. Six pathways were identified (Table 3). Among the 33 gene-pathway relationships identified were ten genes associated with pathways in cancer (*p* value = 1.64*E* − 03), four genes implicated in p53 signaling (*p* value = 2.05*E* − 02), and six genes enriched for cytokine-cytokine receptor interactions (*p* value = 7.38*E* − 02). Although some of the results were associated with *p* values slightly larger than 0.05, they were still included in the table because the fold enrichment was greater than 2.5 for each enriched pathway, which provides supplemental support for their inclusion as they offer insight into the cellular and molecular processes underlying sperm differentiation, activation, and function in the python.

## 4. Discussion

The discovery of python genes associated with sperm phenotypes provides a tremendously important genetic resource for future use in studying reproduction and fertility in endangered and invasive reptile species. The results reported here highlight the value of comparative genomics and its application in species for which genomic resources are available. Through the mapping of python genes to specific phenotypes, it is now possible to develop more focused genetic research projects aimed at identifying genes associated with poor male fertility and reproductive success in endangered populations.

Our analysis of nucleotide and protein sequence similarity between* P. regius* and* P. bivittatus* provides evidence of similarity ranging from 86% to 98% at the nucleotide level and even higher when considering similarity at the protein level. Subsequently each of these two species can serve as a model organism for the other. For example, the genomic resources available for* P. bivittatus* can be used as a model for* P. regius*, such as for applications like PCR primer design. Housley et al. assessed PCR success among cross-species PCR primers and identified the relatedness of the target species and index species as one of the most important factors underlying PCR success [57]. Similarly, genetic dissection of* P. regius* phenotypes can be leveraged for applications in* P. bivittatus*. Unlike* P. bivittatus*,* P. regius* is a much smaller and more docile species which is amenable to reproductive studies as they are easily maintained in captivity and are known for being easy to breed in captivity.

The protein sequences selected to create alignments and phylogenetic trees were based on the limiting number of* P. regius* sequences available in NCBI (fewer than 30 protein sequences). Nonetheless, those selected were all implicated in sperm physiology or related to sperm biology. For example, the mitochondrial protein cytochrome b has been associated with decreased sperm mobility in association with specific haplotypes [58] and mutations in cytochrome b have been linked with asthenospermia [59]. Interestingly, Chen et al. demonstrated that cytochrome b is differentially expressed in X-chromosome versus Y-chromosome containing sperm [60].

The gene encoding epididymal protein E1 participates in sperm physiology and is associated with important sperm parameters. Giacomini et al. identified a threefold decrease in the expression of epididymal protein E1 in oligoasthenozoospermia compared to normozoospermia [61]. Epididymal protein E1 has been identified as a seminal plasma protein across species, such as boars [39] and bulls [62]. Moreover, a study in rams identified this protein as a factor that when added to frozen/thawed semen increased motility through repair of sperm damage that occurred during the cryopreservation process [63]. This protein exhibited marked conservation across taxa in our data. Specifically, it was conserved across mammals, birds, fish, reptiles, and even insects.

The protein cathepsin D is also involved in sperm biology and appears to play a role in its maturation. In mice, cathepsin D expression has been observed in the testis and cathepsin D has been detected on the surface of mouse sperm during epididymal maturation [48]. Similar results have been observed in humans. For example, cathepsin D expression has been observed in human Sertoli cells and Leydig cells and was shown to be anchored to the sperm surface in the postacrosomal region [64]. Evidence that cathepsin D may exhibit an evolutionarily conserved role in sperm physiology comes from a recent study in which cathepsin D was identified as a seminal plasma protein in carp [65].

Based on our multiple sequence alignments across taxa, proteins we investigated each exhibited unique patterns of conservation and divergence across the species. On the one hand, the conservation suggests that these factors have been conserved for millions of years to maintain their specific role in male reproduction. On the other hand, the distinct patterns of divergence between snakes and other reptiles, and even between reptiles and mammals, suggest that the biology may vary between different groups of taxa. Subsequently it is of value to expand our knowledge of the molecular basis of reproduction in reptiles and in particular snakes that can serve as research models for both endangered and invasive species.

In an attempt to expand molecular and genetic knowledge in reptile reproductive biology, we have leveraged a comparative genomics approach to identify* P. bivittatus *genes likely to be associated with sperm phenotypes. Although there are hundreds of genes implicated in reproductive biology, we specifically chose to limit our effort to just those for which each single gene we identified has been previously shown to cause a sperm phenotype in the mouse.

Our rationale for such stringency in our approach was that we prefer to identify a small set of genes, for which the proportion of true positives is very high, compared to a large number of predictions for which the false positive rate is high. When making bioinformatics predictions, one must always consider the trade-off that exists between false negatives and false positives [66, 67]. In our particular case, we wanted to provide a public resource that can facilitate efforts aimed at elucidating reptile reproduction. Since the time and cost of validating bioinformatics predictions in the laboratory are proportional to the number of predictions made, we chose to maximize the number of true positives and subsequently minimize the laboratory cost per true positive identified. The complete set of publicly released data is available in the form of six supplemental files associated with this study.

Although this work leverages bioinformatics and comparative genomics approaches, the genes identified are merely predicted to have sperm related phenotypes in the python species. As evidenced by the phylogenetic analysis performed as part of this study, protein sequences can diverge greatly across taxa. Using mouse genomic annotation to characterize reptile reproductive biology is a challenging process. Nonetheless, the identification of these genes is exciting and provides new avenues of subsequent investigation. However, one must proceed cautiously as it is likely some of the gene-phenotype associations we report may not be present in reptiles. Since it is well known that the reproductive biology of reptiles differs from mammals, the true value of this gene set and the accuracy of the functional predictions will require further study. Even so, the results obtained provide an important first step in expanding reproductive genomics to pythons. Future investigations into these ecologically important species will undoubtedly elucidate a variety of conserved and divergent reproductive properties between reptiles and mammals. Perhaps, some of these discoveries may offer novel pharmacological targets for developing novel reproductive technologies, not only in reptiles, but also in mammals.

## 5. Conclusion

To explore the possibility that* P. regius* and* P. bivittatus *exhibit sufficient genetic similarity to justify using one species as a genetic model for the other species, we assessed the level of sequence identity among a set of protein coding sequences, mitochondrial DNA sequences, and protein sequences between* Python bivittatus *(Burmese python) and* Python regius *(ball python) to assess the extent of sequence identity between the two species in genes implicated in sperm maturation and function. Alignments of epididymal protein E1 demonstrated the molecular similarity between these species with 98% identity when comparing the mRNA and 98% identity when comparing the protein sequences. In multiple sequence alignments and phylogenetic analysis we identify snake specific patterns of sequence similarity in epididymal protein E1 and cytochrome b which support the use of* P. regius* as a model for* P. bivittatus *and other snakes. Most importantly, we employed a comparative genomics strategy to identify python genes enriched for association with sperm related phenotypes. Our approach identified 129 gene-phenotype relationships corresponding to 98 unique genes representing ten specific sperm associated phenotypes. We characterized these genes using gene ontology enrichment to annotate the biological processes, cellular compartments, and molecular functions associated with these reproductively important python genes. Our analysis also identified KEGG pathways enriched for these genes. Specifically, these genes are involved in the regulation of cell cycle, apoptosis, cancer, cytokine-cytokine signaling, and calcium regulation. To our knowledge, our results provide the first comprehensive view of genes associated with sperm development, sperm morphology, and sperm function in pythons. By making our data sets and findings publicly available, in the form of six supplemental files, we hope to facilitate the elucidation of reptile reproduction and promote effective conservation and management of reptiles worldwide.

## Supplementary Material

The authors of this project believe that the benefit of genomics and genetic resources is best accomplished when such resources are freely made available to the research community. Subsequently, the set of mouse and python gene identifiers, along with their orthologous mappings and functional associations with specific phenotypes, have been made freely available to the research community through the supplemental data associated with this publication (Supplemental File 1 and Supplemental File 2). Specifically these supplemental resources include tab-delimited files with gene ontology (biological process, cellular compartment, and molecular function) enrichment results for the sperm associated genes (Supplemental File 3, Supplemental File 4, and Supplemental File 5, resp.) as well as a FASTA file containing the protein sequences for *Python bivittatus* along with the corresponding sperm associated phenotype for each sequence (Supplemental File 6).

## Figures and Tables

**Table 1:** Python orthologs of genes previously associated with sperm function and/or traits along with literature references.

Gene name	*P. regius* (Genbank IDs)	Identity & *e*-value	*P. bivittatus* (Genbank IDs)	Reference
Epididymal secretory protein type E1	gi|698375281 gb|JAC94921.1	99% 8*e* − 114	gi|602671876 ref|XP_007441448.1	[39]

Complement C3	gi|698375324 gb|JAC94937.1	95% 0.0	gi|602634163 ref|XP_007423955.1	[40]

Phospholipase B	gi|698375293 gb|JAC94925.1	96% 0.0	gi|602641944 ref|XP_007427768.1	[41]

L-Amino acid oxidase	gi|698375301 gb|JAC94928.1	N/A	N/A	[42]

Cysteine-rich secretory protein A	gi|698375279 gb|JAC94920.1	95% 1*e* − 166	gi|602662716 ref|XP_007436999.1	[43]

Kallikrein	gi|698375306 gb|JAC94930.1	97% 0.0	gi|602667543 ref|XP_007439332.1	[44]

Factor X	gi|669634856 gb|JAC88993.1	94% 0.0	gi|602632281 ref|XP_007423033.1	[45]

Alpha enolase	gi|5305427 gb|AAD41646.1	88% 0.0	gi|602651461 ref|XP_007432114.1	[46]

Cystatin F	gi|698375284 gb|JAC94922.1	97% 7*e* − 115	gi|602642301 ref|XP_007427941.1	[47]

Cathepsin D	gi|698375257 gb|JAC94912.1	98% 0.0	gi|602659329 ref|XP_007435358.1	[48]

NADH dehydrogenase subunit 4 (mitochondrion)	gi|74310582 ref|YP_313703.1	89% 0.0	gi|511768956 ref|YP_008083608.1	[49]

Cytochrome B (mitochondrion)	gi|74310585 ref|YP_313706.1	94% 2*e* − 63	gi|224815091 gb|ACN65710.1	[50]

ATP synthase F0 subunit 6 (mitochondrion)	gi|74310578 ref|YP_313699.1	90% 5*e* − 126	gi|511768952 ref|YP_008083604.1	[51]

Python orthologs of genes previously associated with sperm function and/or traits along with literature references. Among a set of 21 *P. regius* gene sequences available within the NCBI database, 13 were implicated in sperm function and/or traits based on previous publications in other species. The table contains the *P. regius* NCBI identifiers and corresponding orthologous *P. bivittatus* gene identifiers (for genes in which the ortholog was identified). For each orthologous pair, the protein percent identity and *e*-value obtained from the BLASTP analysis of the sequences are provided. Additionally, each *P. regius* gene identified has a reference implicating the gene in sperm related biology in another species. Together these genes represent evidence that *P. bivittatus *and *P. regius* exhibit high sequence identity among these genes. Specifically, 4 out of the 12 identified ortholog pairs have identity of 97% or greater (epididymal secretory protein E1, Kallikrein, Cystatin F, and cathepsin D), while just two ortholog pairs have percent identity below 90% (alpha enolase having 88% and mitochondrial NADH dehydrogenase subunit 4 with 89%).

**Table 2:** Gene ontology enrichment.

Category	GO identifier	Annotation term	Count	Enrichment	*p* value
Biological process	GO:0019953	Sexual reproduction	45	13.99	7.20*E* − 40
Biological process	GO:0007276	Gamete generation	41	14.78	7.70*E* − 37
Biological process	GO:0048232	Male gamete generation	37	17.11	3.46*E* − 35
Biological process	GO:0007283	Spermatogenesis	37	17.11	3.46*E* − 35
Biological process	GO:0032504	Multicellular organism reproduction	42	12.28	1.54*E* − 34
Biological process	GO:0048609	Reproductive process in a multicellular organism	42	12.28	1.54*E* − 34
Biological process	GO:0003006	Reproductive developmental process	27	14.67	2.70*E* − 23
Biological process	GO:0048610	Reproductive cellular process	22	19.34	2.55*E* − 21
Biological process	GO:0007281	Germ cell development	18	25.38	2.20*E* − 19
Biological process	GO:0048515	Spermatid differentiation	14	34.98	6.97*E* − 17
Biological process	GO:0007286	Spermatid development	13	34.28	1.62*E* − 15
Biological process	GO:0007548	Sex differentiation	15	14.15	2.05*E* − 12
Biological process	GO:0045137	Development of primary sexual characteristics	12	13.46	1.15*E* − 09
Biological process	GO:0008406	Gonad development	11	13.99	5.09*E* − 09
Biological process	GO:0048608	Reproductive structure development	11	12.43	1.60*E* − 08
Biological process	GO:0046661	Male sex differentiation	9	17.56	3.73*E* − 08
Biological process	GO:0051327	M phase of meiotic cell cycle	9	13.08	3.79*E* − 07
Biological process	GO:0007126	Meiosis	9	13.08	3.79*E* − 07
Biological process	GO:0051321	Meiotic cell cycle	9	12.82	4.43*E* − 07
Biological process	GO:0046546	Development of primary male sexual characteristics	7	15.34	5.69*E* − 06
Biological process	GO:0009566	Fertilization	7	12.62	1.77*E* − 05
Biological process	GO:0008584	Male gonad development	6	16.43	2.92*E* − 05
Cellular component	GO:0005929	Cilium	7	8.89	1.24*E* − 04
Cellular component	GO:0031514	Motile secondary cilium	3	122.90	2.13*E* − 04
Cellular component	GO:0042995	Cell projection	14	3.29	2.44*E* − 04
Cellular component	GO:0001669	Acrosomal vesicle	4	17.72	1.41*E* − 03
Cellular component	GO:0019861	Flagellum	4	14.25	2.65*E* − 03
Cellular component	GO:0030141	Secretory granule	6	5.46	4.57*E* − 03
Cellular component	GO:0016023	Cytoplasmic membrane-bounded vesicle	10	2.98	5.65*E* − 03
Cellular component	GO:0031988	Membrane-bounded vesicle	10	2.89	6.93*E* − 03
Cellular component	GO:0005625	Soluble fraction	7	3.66	1.14*E* − 02
Cellular component	GO:0031410	Cytoplasmic vesicle	10	2.55	1.47*E* − 02
Cellular component	GO:0015630	Microtubule cytoskeleton	9	2.69	1.72*E* − 02
Cellular component	GO:0031982	Vesicle	10	2.45	1.89*E* − 02
Cellular component	GO:0000267	Cell fraction	13	1.97	2.79*E* − 02
Cellular component	GO:0033391	Chromatoid body	2	54.62	3.56*E* − 02
Cellular component	GO:0060293	Germ plasm	2	46.82	4.14*E* − 02
Cellular component	GO:0045495	Pole plasm	2	46.82	4.14*E* − 02
Cellular component	GO:0043186	P granule	2	46.82	4.14*E* − 02
Cellular component	GO:0034464	BBSome	2	46.82	4.14*E* − 02
Cellular component	GO:0060170	Cilium membrane	2	40.97	4.72*E* − 02
Molecular function	GO:0046983	Protein dimerization activity	13	3.71	1.60*E* − 04
Molecular function	GO:0042802	Identical protein binding	14	3.38	1.96*E* − 04
Molecular function	GO:0043565	Sequence-specific DNA binding	12	3.06	1.61*E* − 03
Molecular function	GO:0003707	Steroid hormone receptor activity	4	12.62	3.76*E* − 03
Molecular function	GO:0015631	Tubulin binding	5	7.73	3.82*E* − 03
Molecular function	GO:0030554	Adenyl nucleotide binding	20	1.96	4.37*E* − 03
Molecular function	GO:0001883	Purine nucleoside binding	20	1.93	5.16*E* − 03
Molecular function	GO:0042803	Protein homodimerization activity	8	3.70	5.46*E* − 03
Molecular function	GO:0001882	Nucleoside binding	20	1.92	5.55*E* − 03
Molecular function	GO:0004879	Ligand-dependent nuclear receptor activity	4	10.66	6.04*E* − 03
Molecular function	GO:0030528	Transcription regulator activity	19	1.94	6.36*E* − 03
Molecular function	GO:0003700	Transcription factor activity	14	2.22	8.63*E* − 03
Molecular function	GO:0046982	Protein heterodimerization activity	6	4.46	1.06*E* − 02
Molecular function	GO:0005524	ATP binding	18	1.88	1.12*E* − 02
Molecular function	GO:0047485	Protein N-terminus binding	4	8.35	1.18*E* − 02
Molecular function	GO:0032559	Adenyl ribonucleotide binding	18	1.86	1.27*E* − 02
Molecular function	GO:0004672	Protein kinase activity	10	2.55	1.50*E* − 02
Molecular function	GO:0016563	Transcription activator activity	8	3.02	1.58*E* − 02
Molecular function	GO:0004674	Protein serine/threonine kinase activity	8	2.88	2.00*E* − 02

*Gene Ontology Enrichment*. The set of *P. bivittatus* identified as having sperm associated phenotypes were further explored to identify enrichment of gene ontology (GO) terms. A subset of the results are shown above, and the complete set of data is available in Supplemental File 3, Supplemental File 4, and Supplemental 5. Enrichment within the three categories of gene ontology (biological process, cell component, and molecular function) was identified. The displayed GO annotation terms correspond to the most significant *p* values in each of the three categories. The number of genes associated with each annotation term is included, as are the *p* value and the fold enrichment. The enrichment analysis was performed using the DAVID resource. Bonferroni corrected *p* values and false discovery rate values are included in the tab-delimited supplemental files as well as the list of genes associated with each GO annotation term.

**Table 3:** Identification of KEGG pathways enriched for genes within *Python bivittatus* genes associated with sperm phenotypes.

Pathway-ID	Pathway name	Count	Genes	Enrichment	*p* value
hsa05200	Pathways in cancer	10	LAMA2, FOS, AR, PDGFA, RXRB, BAX, NOS2, KIT, FAS, CCNA1	3.45	1.64*E* − 03
hsa04115	p53 signaling pathway	4	BAX, APAF1, FAS, ATM	6.65	2.05*E* − 02
hsa05222	Small cell lung cancer	4	LAMA2, RXRB, APAF1, NOS2	5.38	3.54*E* − 02
hsa04210	Apoptosis	4	BAX, APAF1, FAS, ATM	5.20	3.87*E* − 02
hsa04020	Calcium signaling pathway	5	ATP2B4, CAMK4, PLCD4, NOS2, BDKRB2	3.21	6.44*E* − 02
hsa04060	Cytokine-cytokine interaction	6	LEP, AMHR2, AMH, PDGFA, KIT, FAS	2.59	7.38*E* − 02

Identification of KEGG pathways enriched for genes within *Python bivittatus* genes associated with sperm phenotypes. KEGG pathways were identified using the bioinformatics resource DAVID. The KEGG pathway identifier and pathway name are provided along with the number of genes identified in the pathway, the gene symbol for each identified gene, the fold enrichment, and the associated *p* value.
